# Exploring the role of empathy in nurses’ intention to provide disaster care: a cross-sectional study

**DOI:** 10.1186/s13584-025-00721-4

**Published:** 2025-09-22

**Authors:** Anna Goudetsidou, Theodoros Pesiridis, Petros Galanis, Venetia Sofia Velonaki

**Affiliations:** Faculty of Nursing, National and Kapodistrian University of Athens, Athens, Greece

**Keywords:** Nurses, Disaster nursing, Emergency events, Intention, Willingness, Theory of planned behavior, Empathy

## Abstract

**Background:**

Over the past two decades, the increasing frequency of disasters highlights the urgent need for nurses willing to respond to these crises. Although disaster care is part of their professional role, willingness to provide care in such situations is not always guaranteed and may be influenced by various factors. Understanding what shapes their intention to provide care is critical for effective disaster planning. This study investigates the role of empathy in shaping nurses’ intention to participate in disaster care, drawing on the Empathy-Altruism Hypothesis. To further contextualize this relationship, elements of the Theory of Planned Behavior—attitude toward the behavior, subjective norms, and perceived behavioral control—were also examined as potential predictors of behavioral intention.

**Methods:**

A cross-sectional and correlational study was carried out among nurses in Greece using a convenience sampling method. Data collection took place from December 2023 to April 2024. Participants completed a questionnaire covering demographics, academic background, experiences with disasters, empathy (assessed using the Jefferson Scale of Empathy), and behavioral intentions related to disaster care. The Behavioral Intention Scale was used to assess the behavioral intention, attitude toward the behavior, subjective norms and perceived behavioral control.

**Results:**

The final sample included 252 nurses. Findings revealed a strong intention to participate in disaster victim care. Empathy levels were moderate to high, with a mean score of 103.56 on a 20–140 scale. Multiple linear regression, using behavioral intention as the dependent variable, revealed a significant positive association between subjective norms and behavioral intention. In contrast, empathy, attitude, and perceived behavioral control were not significantly associated with behavioral intention.

**Conclusions:**

This study provides insights into the factors that influence nurses’ intention to provide disaster care, with subjective norms emerging as the strongest predictor. These findings partially support the Theory of Planned Behavior and suggest that social expectations and perceived peer support may be critical in shaping willingness to respond. Although nurses exhibited strong empathy and a high intention to participate in disaster care, empathy did not significantly influence behavioral intention. Further research is needed to explore empathy’s potential contribution to motivating nurses to engage in disaster response.

## Background

From ancient times to the present day, disasters have been an integral part of life. People have tried to explain and express their concern about disasters through art, religion, and mythology. According to The International Disaster Database (EM-DAT), between 2000 and 2019, the emergency events database recorded 7,348 natural disaster events worldwide. These disasters resulted in approximately 1.23 million deaths and affected over 4 billion people. This marks a significant increase compared to the previous twenty years (1980–1999), during which 4,212 natural disasters were recorded, causing approximately 1.19 million deaths and affecting more than 3 billion people [1]. The increasing frequency and impact of disasters underscore the critical need for nurses who are both willing and adequately prepared to respond effectively during such events [2].

Addressing all health needs that emerge during a disaster necessitates the extensive involvement of nurses. However, several studies have reported that healthcare professionals’ willingness to respond during disasters is not consistently high and often fluctuates depending on various circumstances. Choi et al. found that only 21% of emergency nurses were willing to respond to a radiological event [3]. Al-Hunaishi et al. reported that while overall willingness was high, it declined in pandemic-related scenarios, highlighting the importance of perceived risk and confidence [4]. Furthermore, an earlier study showed that healthcare workers’ willingness to report to duty during catastrophic events ranged from 48% to 85% of participants, depending on various factors [5]. Type of disaster, concern for personal safety and perceived self-efficacy were identified as factors that either supported or limited healthcare professionals’ willingness to respond to an emergency event [6–8]. Understanding the proportion of nurses willing to engage in crisis and disaster management, along with the factors influencing their decisions, is essential for the effective development and implementation of disaster response plans.

Behavioral models and theories have been applied to explore the factors that are related to healthcare workers’ willingness to participate in a disaster. A widely utilized framework for examining the behavioral intention of healthcare workers is derived from Ajzen’s Theory of Planned Behavior (TPB). This framework has been broadly applied in health-related research to examine factors influencing professionals’ willingness to engage in challenging clinical contexts [9–12]. According to the TPB, the intention that drives behavior can be predicted by three factors: the individual’s attitude toward the behavior, subjective norms, and the perceived behavioral control. Specifically, attitude toward the behavior refers to the extent to which an individual evaluates a behavior as favorable or unfavorable. In other words, if nurses believe that participating in the care of disaster victims is important and will lead to positive outcomes, they are likely to develop a positive attitude toward this behavior. Subjective norms refer to the perceived social pressure to engage or not engage in a particular behavior. If a nurse believes that their family, colleagues, or hospital management, expect them to respond to disasters and consider it a duty, they are more likely to intend to do so. Perceived behavioral control refers to an individual’s perception of how easy or difficult it is to perform a particular behavior. It reflects the extent to which a person feels they have control over the behavior, considering various factors (e.g. skills, knowledge, confidence, resources, and obstacles) [13]. For example, a nurse who feels well-prepared and knowledgeable about disaster response will have a higher perceived behavioral control than one who feels unprepared and finds it difficult to do so.

Another factor that may play a role in shaping healthcare workers’ intention to participate in a disaster response is empathy. Empathy demonstrated by health professionals in disaster settings plays a crucial role in alleviating victims’ concerns and uncertainties [14]. During the COVID-19 pandemic, research underscored the importance of empathy in effectively communicating public health information and protective measures [15]. Additionally, a study exploring the relationship between empathy and nursing students’ intention to care for individuals with HIV revealed a positive correlation between these variables [16]. Similarly, research on nursing students demonstrated a positive relationship between empathy and their intention to provide care to elderly individuals, showing that as empathy increased, so did their willingness to offer care [17]. The Empathy-Altruism Hypothesis suggests that empathy - the ability to understand and share the feelings of another - can lead to altruistic behavior - actions that are motivated by a genuine desire to help others [18]. According to this hypothesis, empathy is not merely an emotional response but a motivational force that can lead to altruistic behavior, especially toward vulnerable populations. In the context of disaster response, this theory may help explain why nurses choose to provide care despite significant personal risks, such as exposure to trauma and hazardous conditions. Victims of disasters often experience acute emotional, psychological, and physical trauma, and nurses who perceive this suffering through an empathic lens may feel a heightened sense of responsibility and urgency to act. The empathy-based desire to relieve human suffering can override personal discomfort or fear, influencing nurses’ decision-making and increasing their behavioral intention to respond in catastrophic events.

Building on this theoretical framework, our study aims to explore the association between empathy and nurses’ intentional behavior in disaster settings. Specifically, it investigates how empathy might motivate nurses to engage in healthcare for disaster victims, even when such care requires personal sacrifice or involve significant challenges. Despite the growing body of literature on empathy in healthcare, there are no studies that have examined the impact of empathy—whether positive or negative—on nurses’ intention to participate in disaster-related healthcare, which presents a gap that this study seeks to address.

## Method

### Aim and study design

This cross-sectional survey and correlational study were conducted to explore nurses’ intention to provide care during catastrophic events and the potential role of empathy in shaping this intention. Specifically, the study sought to:

1. Assess the proportion of nurses working in Greece who are willing to provide care after a mass disaster,
2. Identify the factors that are related to their intention to do so,
3. Evaluate the level of empathy among nurses practicing in Greece, and
4. Examine the relationship between empathy and behavioral intention.

To achieve these aims, a convenience sample was used that covered the diversity of nurses who work in Greece. We approached registered nurses from various facilities including hospitals, schools, primary health settings and workplaces with occupational nurses. The inclusion criteria for the sample were as follows: participants had to provide informed consent and have worked as nurse in Greece for at least six months. Exclusion criteria included nurse assistants and individuals who declined to participate. For hospital nurses, the survey was distributed in person; the researchers visited the hospitals and invited nurses to participate voluntarily and anonymously. For school nurses, occupational nurses, and nurses in primary healthcare settings, the questionnaire was administered online via Google Forms to colleagues working in these settings. Ethical approval was granted by the Ethics Committee of the Department of Nursing at the National and Kapodistrian University of Athens and the hospital scientific councils.

## Survey measures (self-report)

The final questionnaire included 46 questions and consisted of the following sections:

- Socio-demographic variables included gender, age, marital status, number of children in the family, place of residence, educational status, years of work experience and work facility.
- Disaster-related questions covering disaster education, work experience in disasters, personal experience with disasters, and concerns about future disasters.
- Behavioral Intention (BI): Nurses’ BI and its predictors were assessed using the BI scale, constructed according to Ajzen’s Theory of Planned Behavior [13]. This scale includes twelve items: three for generalized behavioral intention, the dependent variable (e.g., “I intend to participate in providing care to victims of a major disaster, should I be called upon”), three for subjective norms (e.g., “People who are important to me would support my decision to provide care to individuals affected by a major disaster”), three for attitudes (e.g., “I believe that nurses’ participation in caring for individuals affected by disasters or mass casualty incidents is important”), and three for perceived behavioral control (e.g., “The decision to participate in providing care to victims of a major disaster depends solely on me and no one else”). Responses were recorded using a 7-point Likert-type scale, with endpoints adapted to the content of each item. The questionnaire is based on an older version [2] and has been tested for reliability (internal consistency α = 0.57–0.93). In this study Cronbach’s alpha for Behavioral Intention scale was calculated to be 0.823.
- Nurses’ empathy: the Jefferson Scale of Empathy (JSE) – (Health Professional Version) was used to assess nurses’ empathy, with permission from Thomas Jefferson University (© Thomas Jefferson University, 2001 All rights reserved). This scale, developed by Mohammadreza Hojat [19], includes 20 questions rated on a 7-point Likert scale, where 1 corresponds to “strongly disagree” and 7 to “strongly agree”. It was translated into Greek using the back-translation method and its psychometric properties were evaluated in relevant Greek populations. In a study involving dental postgraduate students, the internal consistency of the scale was found to be satisfactory, with a Cronbach’s alpha of 0.76 and an average item discrimination index of 0.33 [20]. In a separate study conducted among Greek nurses, the reliability of the Greek version was further supported through test–retest analysis, which yielded an intraclass correlation coefficient (ICC) of 0.876 (*p* < 0.0001), indicating strong temporal stability. Internal consistency was also adequate, with Cronbach’s alpha coefficients of 0.796 and 0.734 at two different time points [21]. In this study Cronbach’s alpha for Jefferson Scale of Empathy was calculated to be 0.860.

## Response rate

The required sample size was calculated using G*Power 3.1.9.2 [22]. Based on a small effect size (f² = 0.06), a power level of 0.95, an alpha error probability of 0.05, and 17 independent variables, the required sample size for a linear multiple regression was estimated at 219 participants. To meet this requirement, we distributed the questionnaire to 340 registered nurses working in two public hospitals, primary healthcare settings, schools or as occupational nurses, in different workplaces in Greece. A total of 269 completed questionnaires were returned, with a study response rate of 79.11%. Of these, 17 questionnaires were deemed invalid and were not included in the statistical analysis. Therefore, the analysis and presentation of results pertain to 252 nurses.

### Statistical analysis

Continuous variables are expressed as mean, standard deviation, median, minimum value, and maximum value, while categorical variables are shown as absolute counts (n) and relative frequencies (%). We used the Kolmogorov-Smirnov test to check the normality of the distribution of the Behavioral Intention scale and Jefferson Scale of Empathy. The results indicated that both variables were normally distributed. To assess differences between independent variables and the behavioral intention score, independent samples t-tests, Pearson’s correlation coefficient, and Spearman’s correlation coefficient were applied. Then, we used multivariate linear regression including all independent variables, with behavioral intention score as the dependent variable. Statistical analysis was performed with the Statistical Package for Social Sciences for Windows, Version 22.0 with a 2-tailed significance level of 0.05 and 95% confidence interval.

## Results

### Socio-demographic characteristics

Among the 252 nurses, most were female (77.4%) and the average age was 40.6 (SD = 10.9). Just over half of the participants were married or in a permanent relationship (51.6%), while the same percentage reported having no children (51.6%). The nurses who participated in the study were mainly residents of a city or suburb (96%), while a small percentage lived in a small town, rural area or village (2%). Slightly less than half of the participants had a master’s degree (43.6%) and a small proportion held a doctorate (3.6%). The average length of service in the health field was 15.62 years, and the majority (84.9%) of the nurses worked in a hospital. Demographic and job characteristics of the participants are shown in Table 1

**Table 1:** Demographic and job variables (*N* = 252)

Variables	*n* (%)
*Gender*	
Male	56 (22.2)
Female	195 (77.4)
No answer	1 (0.4)
*Age*	
Mean	40.62
Std. Deviation	10.93
Minimum	22
Maximum	62
No answer	7 (2.8)
*Marital status*	
Single	104 (41.3)
Married/Permanent relationship	130 (51.6)
Divorced	16 (6.3)
Widowed	2 (0.8)
*Children*	
0	130 (51.6)
1	38 (15.1)
2	69 (27.4)
3	14 (5.6)
≥ 4	1 (0.4)
Residence	
Rural area/village	5 (2.0)
Small town	5 (2.0)
City/suburb of city	242 (96)
*Educational level*	
Master	110 (43.6)
PhD	9 (3.6)
Bachelor	133 (52.8)
*Work experience (years)*	
Mean	15.62
Std. Deviation	11.30
Minimum	0.5
Maximum	42
No answer	1
*Work facility*	
Hospital	214 (84.9)
Primary healthcare settings	20 (7.9)
School	15 (6.0)
Occupational nurses	3 (1.2)

## Disaster-related characteristics

Seventy-seven-point 8% (77.8%) of the participants stated that they had not received any disaster training, while nearly two-thirds (62.7%) had professional experience as nurses in disaster situations. The majority of participants (72.2%) reported experiencing no impact or only minor impact on their personal lives from a disaster, while 16.7% reported that they experienced some injury, loss, or material damage to themselves or a relative due to a catastrophic event. Finally, a large portion of participants (60.8%) reported moderate, high, or very high levels of worry about the possibility of a disaster occurring in their area. The results are shown in Table 2.

**Table 2:** Disaster-related variables (*N* = 252)

Variables	*n* (%)
*Disaster training*	
No	196 (77.8)
Yes	56 (22.2)
*Professional experience in disasters*	
No experience	94 (37.3)
Limited experience	70 (27.8)
Moderate experience	52 (20.6)
Strong experience	22 (8.7)
Extensive experience	14 (5.6)
*Personal impact from a disaster*	
No impact	101 (40.1)
Minor impact	81 (32.1)
Moderate impact	37 (14.7)
Significant impact	25 (9.9)
Profound impact	8 (3.2)
*Injuries/Loss/Material damage*	
Yes	42 (16.7)
No	210 (83.3)
*Worry about a future disaster*	
None	23 (9.1)
Low	76 (30.1)
Moderate	74 (29.4)
High	35 (13.9)
Very high	44 (17.5)

## Behavioral intention and empathy

Scores on the Behavioral Intention scale range from 3 to 21, with a midpoint of 12. Higher scores indicate a greater behavioral intention to provide care to disaster victims. Out of a possible 21 points, respondents scored high with a mean of 17.81 (SD = 3.15) points. According to the BI scale, the vast majority of participants (90.5%) believe that they would provide care to victims, if called upon after a disaster. A total of 83.8% of participants stated that they would like the opportunity to provide care after a disaster. Additionally, 84.6% of nurses expressed their intention to participate in providing care after a disaster, if called upon.

The total score on the Jefferson Scale of Empathy ranged from 20 to 140. Higher scores indicate greater level of empathy. Respondents scored a mean of 103.56 (SD = 14.8) points indicating moderate to high levels of empathy.

### Bivariate analysis

Table 3 presents the correlations between continuous variables and the behavioral intention (dependent variable). Pearson’s correlation coefficients were used, except for work experience (years), where Spearman’s correlation was applied due to non-normal distribution. Independent-samples t-tests were conducted to examine differences in behavioral intention scores across dichotomous groups (Table 4). According to bivariate analysis, none of the socio-demographic characteristics was statistically significantly associated with behavioral intention. However, disaster-related education (e.g., seminars or postgraduate study programs) appears to be associated with a higher behavioral intention score (*p* = 0.038). Additionally, worry about a future disaster in the participants’ place of residence was positively associated with behavioral intention (*p* = 0.044). In other words, the greater the worry, the higher the score on the intention to provide disaster care. No statistically significant relationship with behavioral intention was found for professional experience in disasters, personal experience, injury, losses, or material damage from a catastrophic event. Subjective norms, attitudes, and perceived behavioral control are positively correlated with behavioral intention. In other words, as the scores for these three predictor variables increase, the intention to provide care to those affected by a disaster also increases. Moreover, empathy is positively correlated with nurses’ behavioral intention. Specifically, higher empathy scores are associated with a greater intention among nurses to provide care to disaster victims.

**Table 3:** Correlations between continuous variables and behavioral intention (dependent variable)

Independent Variables	Mean (Std. Deviation)	Correlation Coefficient	*p*-value
*Subjective norms*	17.15 (3.19)	**0.699**	**< 0.001**
*Attitudes*	17.81 (3.61)	**0.147**	**0.019**
*Perceived behavioral control*	12.43 (2.72)	**0.253**	**< 0.001**
*Empathy*	103.56 (14.80)	**0.255**	**< 0.001**
*Age*	40.62 (10.93)	0.014	0.830
*Work experience (years)*	15.62 (11.30)	−0.013	0.835

Values in bold indicate statistical significance at *p* < 0.05

**Table 4:** Bivariate analysis between independent variables and the behavioral intention (dependent variable)

Independent Variables	Mean (Std. Deviation)	*p*-value
*Sex*		0.240
Male	17.33 (3.52)	
Female	17.95 (3.03)	
*Marital status*		0.519
Married/Permanent relationship	17.93 (2.83)	
Single/Divorced/Widowed	17.67 (3.46)	
*Children*		0.977
1/2/3/≥4	17.81 (3.00)	
0	17.80 (3.29)	
*Residence*		0.614
Rural area/village/town	18.30 (2.35)	
City/suburb of city	17.78 (3.18)	
*Educational level*		0.421
Master/PhD degree	17.97 (3.24)	
No postgraduate studies	17.65 (3.07)	
*Work facility*		0.062
Hospital	17.64 (3.22)	
School/Occupational nurses/Primary healthcare settings	18.68 (2.59)	
*Disaster training*		**0.038**
Yes	18.50 (2.66)	
No	17.60 (3.25)	
*Professional experience in disasters*		0.619
No/Limited experience	17.87 (3.10)	
Moderate/Strong/Extensive experience	17.67 (3.25)	
*Personal impact from a disaster*		0.654
No/Minor/Moderate impact	17.84 (3.16)	
Significant/Profound impact	17.57 (3.08)	
*Injuries/Loss/Material damage*		0.387
Yes	18.19 (3.52)	
No	17.72 (3.07)	
*Worry about a future disaster*		**0.044**
None/Low	17.30 (3.23)	
Moderate/High/Very high	18.13 (3.06)	

Values in bold indicate statistical significance at *p* < 0.05

### Multivariable linear regression

Bivariate analysis revealed that six independent variables were significantly associated with nurses’ intention to participate in providing care to disaster victims. Therefore, multivariable linear regression was applied, with the results shown in Table 5. According to the analysis, only subjective norms were found to be a statistically significant predictor of behavioral intention (*p* < 0.001). Attitude toward the behavior (*p* = 0.062) and perceived behavioral control (*p* = 0.424) did not reach statistical significance. Furthermore, empathy, which had demonstrated a statistically significant positive correlation with behavioral intention in the bivariate analysis, was not a significant predictor in the multivariable model (*p* = 0.350). These results suggest that, in this sample, behavioral intention was more strongly influenced by subjective norms than by personal attitudes, perceived control, or empathy levels. The model was statistically significant (*p* < 0.001), explaining 52% of the variance in behavioral intention (R² = 0.52, adjusted R² = 0.49). The predictive power of the model is primarily attributable to the research variables rather than to socio-demographic characteristics, a finding confirmed by a two-step (hierarchical) linear regression, which showed a ΔR² = 0.477, F change (6, 229) = 38.268, *p* < 0.001.

**Table 5:** Multivariable linear regression analysis with the behavioral intention score as the dependent variable

Independent Variables	b	95% Confidence interval	*p*-value
Disaster training	0.080	−0.12–1.31	0.105
Worry about a future disaster	−0.004	−0.63–0.58	0.939
Subjective norms	**0.662**	**0.55–0.75**	**< 0.001**
Attitudes	0.092	−0.004–0.16	0.062
Perceived behavioral control	0.041	−0.07–0.16	0.424
Empathy	0.049	−0.01–0.03	0.350

*R* = 0.72, R^2^ = 0.52, Adj R^2^ = 0.49

Values in bold indicate statistical significance at *p* < 0.05

## Discussion

This study primarily examined the relationship between empathy and nurses’ intention to provide care during disasters, drawing on the Empathy-Altruism Hypothesis. Constructs from the TPB—attitude, subjective norms, and perceived behavioral control—were also investigated as additional predictors of intention. Interestingly, while empathy was positively correlated with intention in the bivariate analysis, it was not a significant predictor in the multivariate model. This discrepancy between the bivariate and multivariate findings may suggest that empathy does not exert a direct influence on behavioral intention, but may instead act indirectly through one or more constructs of the Theory of Planned Behavior. For instance, empathy might enhance their perception of social responsibility, which in turn could influence their intention to act. However, the primary aim of the present study was to explore the direct relationship between empathy and behavioral intention. This potential indirect pathway represents an important avenue for future research. An alternative explanation could be the strong influence of subjective norms in this study. In high-pressure, resource-limited settings such as disaster response, nurses’ intentions may be more strongly driven by perceived social expectations—whether from colleagues, supervisors, or the public—than by personal emotional traits like empathy. As a result, the effect of empathy may be diminished or masked when multiple factors are analyzed together.

The present study found that nurses exhibited moderate to high empathy levels, with a mean score of 103.56 (maximum = 140), consistent with a 2019 Greek pilot study where nurses scored 99.18 and 98.37 in two measurements [21]. Literature highlights empathy as a crucial skill for healthcare professionals, which can be effectively enhanced through well-structured educational interventions in academic and clinical settings [23–25]. While our findings demonstrate a positive bivariate association between empathy and nurses’ behavioral intention to respond to disasters, it is important to acknowledge that empathy is a complex construct. According to a recent systematic review, although empathy is generally beneficial, it can also be positively associated with emotional exhaustion. High empathy levels may lead to emotional fatigue in demanding healthcare environments [26]. Additionally, while empathic concern and perspective-taking promote prosocial actions, personal distress is negatively associated with such behavior. These findings suggest that individuals who experience high levels of empathy may be more susceptible to personal distress, burnout and finally less inclined to engage in supportive or helping behaviors, even in the case of a disaster [27]. Therefore, while empathy may initially motivate nurses to respond, excessive emotional involvement could potentially undermine their long-term capacity to cope with the psychological demands of disaster care.

Bivariate analysis revealed that subjective norms, attitudes, and perceived behavioral control were positively correlated with nurses’ intention to participate in the care of victims of catastrophic events. Specifically, as these three predictor variables increased, participants’ behavioral intention also increased. This finding aligns partially with the TPB, which suggests that intention is influenced by attitudes, subjective norms, and perceived behavioral control. However, in the multivariate model, only subjective norms remained a statistically significant predictor (*p* < 0.001). This may indicate that, although attitudes and perceived behavioral control are related to intention when examined independently, their predictive power diminishes when considered alongside subjective norms. This could reflect the particularly strong influence of perceived social expectations—such as approval from colleagues, family, or institutional leadership—on nurses’ willingness to respond in disaster situations. Attitudes toward the behavior did not reach statistical significance, suggesting that viewing disaster response as valuable or important may not, by itself, be sufficient to influence intention. Likewise, perceived behavioral control was not significantly associated with intention, possibly reflecting uncertainty or a lack of confidence among nurses regarding their ability to respond effectively in high-stress conditions. A similar conclusion was reached in the study by Pesiridis et al., which found that subjective behavioral norms, perceived control, and nurses’ knowledge about disasters contributed to an increased intention to provide care to disaster victims [2].

It is encouraging that the nurses in this study demonstrated a strong intention to provide care to disaster victims. The majority of participants expressed their willingness to offer care if called upon in the event of a disaster. The mean value of the intention index was 17.81 (ranging from a minimum of 3 to a maximum of 21), which, according to the TPB, strongly suggests that this intention is likely to translate into actual behavior if the need arises [28]. Pesiridis et al. (2015) also recorded high behavioral intention among nurses to provide healthcare during disasters, with an average value of 17.65 [2]. Consistent findings were observed in the study by Pesiridis et al. (2021), where the intention index of healthcare professionals in Greece to provide care for COVID-19 patients had a mean value of 18.2 [9]. Equally encouraging results were observed in a study conducted in Yemen by Al-Hunaishi et al. (2019), which investigated the intention of 692 healthcare professionals to participate in disaster management and identified the factors influencing it. In that study, 90% of participants expressed a strong intention to engage in disaster management, though this intention was lower for specific scenarios, with 77.3% willing to respond to natural disasters and 66% to pandemics [4]. In the study by Choi et al. [3] involving 158 emergency room nurses in South Korea, participants showed a high intention to respond to natural disasters but significantly lower willingness to respond to technological disasters such as a bomb explosions, chemical attacks or bioterrorism. Specifically, 68% and 65% of nurses were willing to provide services during earthquakes and floods, respectively, while only 21% and 43% were willing to respond to radiological or chemical incidents [3]. This lower willingness to respond to technological disasters may be due to their ambiguous and unpredictable nature, and worries about inadequate resources which often generates fear and safety concerns among healthcare professionals [29, 30]. The higher rate of behavioral intention observed in the present study may be attributed to its focus on nurses’ willingness to respond to any type of disaster, rather than specific events.

Τhe study found that only 22.2% of nurses had previously undergone any form of disaster-related training or victim management, despite the majority expressing significant concern about the potential occurrence of a disaster in their area. This is likely due to the fact that most undergraduate nursing programs in Greece do not include mandatory disaster management courses. The deficiency in knowledge and training among healthcare professionals in Greece, particularly nurses, is further highlighted by similar studies, which assessed the disaster preparedness of healthcare personnel [2, 31]. In this study a minority of nurses (37.3%) reported having no experience in providing care to disaster victims. This finding, along with the small percentage of nurses educated in disaster management, highlights the urgent need for disaster management training, as a significant proportion of nurses will likely need to care for disaster victims at some point in their careers. Bivariate analysis revealed that nurses with prior training in disaster management and victim care were significantly more willing to participate in responding to such events (*p* = 0.038). However, this relationship did not persist in the multivariate model. One possible explanation is that the effect of training may be mediated or diminished when examined alongside stronger psychosocial factors—such as subjective norms—which emerged as the sole significant predictor. In other words, perceived social expectations from peers, institutional leadership, or family may play a more decisive role in shaping behavioral intention than the mere presence or absence of training. It is also likely that variations in the type, quality, or perceived adequacy of training received by participants limited its impact when other variables were simultaneously considered. Nonetheless, it is important to note that a substantial body of literature supports the positive role of disaster education in enhancing healthcare professionals’ willingness to respond during emergencies. Several studies have shown that targeted training improves the intention to respond in disasters [2–4, 32, 33]. These findings suggest that education may not always function as a direct predictor but can still play an important role.

In terms of demographic characteristics, most participants were female nurses with an average age of approximately 40. None of the socio-demographic characteristics showed a statistically significant association with behavioral intention. Gender is one of the factors with inconsistent findings. Some studies have identified a positive correlation between male gender and the intention to provide care during disasters and crisis [34–37], while others have found no correlation [9, 32]. In our study, this lack of association may be partly explained by the gender imbalance in the sample, as the vast majority of participants were female (77.4%), which may have limited the ability to detect significant gender differences. Further research with larger and more diverse samples is warranted to better understand the influence of demographic factors on intentional behavior. Although the work setting (hospital or community) appeared to influence nurses’ intention to participate in disaster care, the association was not statistically significant. A study by Goodhue et al. (2012) found that nurses in emergency departments were more willing to respond to disaster events compared to those in primary care [35].

Although this study was conducted in Greece, its findings have relevance for other countries with similar healthcare system characteristics and disaster vulnerabilities, including Israel. Greece has a publicly funded healthcare system, primarily delivered through the National Health System, which provides universal coverage to all citizens. However, it faces persistent challenges such as limited funding, staff shortages, and organizational inefficiencies—factors that can impair disaster response capacity. Both Greece and Israel have experienced various forms of natural and man-made disasters and in both contexts, nurses play a critical frontline role during crises. Yet formal disaster preparedness training is often limited or inconsistently implemented. For instance, a study conducted in Israel in 2014 concluded that the level of perceived knowledge regarding nurses’ roles in large-scale emergencies was low, underscoring the need for enhanced training initiatives [38]. More recently, a 2025 study by Avraham et al. reported that prehospital emergency nurses in Israel identified gaps in their knowledge and understanding of their roles and duties during mass-casualty incidents, reinforcing the call for targeted education and policy reforms to improve preparedness [39]. These shared vulnerabilities emphasize the importance of developing comprehensive disaster preparedness programs within nursing education and practice. Moreover, while empathy did not emerge as a significant predictor in multivariate analysis, it remains a critical professional quality whose impact may vary across sociocultural settings. The present study’s findings—particularly the influence of subjective norms—may inform policy strategies aimed at strengthening nurses’ preparedness not only in Greece, but also in Israel and other international settings with comparable healthcare and disaster response frameworks.

### Health policy recommendations

Based on the findings of this study and the existing literature, several policy recommendations emerge to enhance disaster nursing preparedness and response:

- To enhance nurses’ preparedness for crisis situations, it is recommended that structured disaster preparedness modules be integrated into core undergraduate nursing education. This could be implemented cost-effectively by utilizing existing faculty and incorporating blended learning approaches (e.g., online lectures, simulations, and workshops). Collaboration between nursing departments and professional bodies would be essential to ensure feasibility and long-term sustainability. This recommendation aligns with international guidelines by organizations such as the World Health Organization (WHO) and International Council of Nurses (ICN), which emphasize disaster readiness as a core nursing competency [40, 41]. For practicing nurses, the Ministry of Health could partner with hospitals and professional nursing associations to offer low-cost or subsidized postgraduate training and certification programs—preferably delivered via online platforms to maximize accessibility to continuing education and certification in disaster response.
- Given the significant impact of subjective norms (family and peer influence), interdisciplinary disaster drills including doctors, emergency medical technicians, and hospital administrators should be conducted to enhance collective preparedness. These can be scheduled as part of routine hospital preparedness exercises to limit additional resource demands.
- Develop clear national and institutional protocols for nurses’ roles in disaster response through collaboration between the Ministry of Health, regulatory bodies, and hospital leadership. Templates from EU guidelines or WHO frameworks can be adapted to reduce development time and cost.

### International implications and generalizability of findings

The influence of subjective norms suggests that nurses’ intention to participate in disaster care is not only an individual decision but also is shaped by the broader professional and societal context. The generalizability of findings in other healthcare systems should be considered cautiously due to the use of a convenience sample. However, the results may be relevant to countries where family and social environments play a key role in shaping people’s choices and behaviors. Therefore, efforts to enhance nurses’ disaster response intentions could benefit from approaches that emphasize the role of social influence and perceived approval from significant others, rather than relying solely on personal motivation.

### Future research

One of the key questions in disaster nursing is how to strengthen healthcare professionals’—particularly nurses’—intention to respond to disasters and provide care to victims. This study examined factors that may influence such intention and introduced the investigation of empathy as a potential contributor to disaster care—a topic not previously studied either in Greece or internationally. Future research should build on the present study to further explore the relationship between empathy and nurses’ behavioral intention to provide care during disasters. Although empathy was not significantly associated with intention in the multivariate analysis, its positive correlation in the bivariate analysis suggests a potential indirect role, which could be explored further—possibly through mediation models involving TPB constructs. Intervention-based studies are strongly recommended, as they can evaluate the effectiveness of structured educational or training programs aimed at enhancing empathy and assessing their subsequent impact on behavioral intentions. Such studies would provide critical insights into whether empathy can be intentionally cultivated to increase nurses’ readiness to respond in crisis situations. Future studies could also use longitudinal approaches to observe how nurses’ behaviors evolve over time and along with qualitative approaches—such as interviews or focus groups—to gain a richer understanding of nurses’ motivations, perceived duties and barriers. Finally, exploring how subjective norms vary across different cultural and healthcare settings could improve our understanding of the social factors shaping disaster care intentions in diverse contexts.

### Limitations

This study used a convenience sample of nurses based on voluntary participation, which may have introduced selection bias. Nurses who chose to take part voluntarily might have held a greater interest in disaster preparedness or positive attitude toward disaster response. This self-selection limits the generalizability of the findings, as the sample may not fully reflect the broader nursing population.

Due to time and resource limitations, most participants were from two general hospitals in Attica, with limited representation from rural or community-based healthcare settings. Although the results aligned with previous Greek study [2] that used systematic random sampling, caution is still warranted when extrapolating the findings.

An additional limitation is the cross-sectional design of the study, which restricts the ability to draw conclusions about causality or to predict actual behavior in disaster scenarios. While behavioral intention is used for predicting future actions, it does not guarantee that nurses would actually engage in disaster response when the situation arises. Factors such as emotional responses, availability of resources, organizational culture, and real-time decision-making constraints can significantly influence actual behavior, beyond what is captured in a questionnaire.

Nurses’ behavioral intention was assessed using a self-report questionnaire, which raises concerns about social desirability bias, as participants might provide socially desirable responses. Nurses might have overreported their willingness to participate in disaster care to appear as “good” nurses. Although anonymity was preserved to mitigate this effect, the potential for response bias cannot be fully excluded.

## Conclusions

The findings of this study elucidate the factors of nurses’ intention to respond during disasters. Although empathy was positively associated with nurses’ intention to provide care to disaster victims in the bivariate analysis, this relationship was not retained in the multivariate model, suggesting that other factors may play a stronger predictive role. In particular, subjective norms emerged as the most significant predictor of behavioral intention, underscoring the importance of social approval and peer influence in motivating nurses’ intention to respond in disaster situations. Nonetheless, empathy’s positive correlation in bivariate findings and its theoretical relevance suggest that it may still play an important role. Further research is warranted to determine whether empathy can be a meaningful factor in enhancing nurses’ willingness to engage in disaster response.

From a policy perspective, efforts to improve disaster response capacity should include not only educational initiatives aimed at building knowledge and skills, but also strategies to leverage social influence, promote positive norms, and foster a culture of readiness within healthcare institutions.

## Data Availability

The data analyzed in this study can be obtained from the author upon reasonable request.

## Abbreviations

TPB: Theory of planned behavior
BI: Behavioral intention
